# Semisynthesis of Derivatives of Oleanolic Acid from *Syzygium aromaticum* and Their Antinociceptive and Anti-Inflammatory Properties

**DOI:** 10.1155/2016/8401843

**Published:** 2016-06-13

**Authors:** Sibusiso Rali, Opeoluwa O. Oyedeji, Olukayode O. Aremu, Adebola O. Oyedeji, Benedicta N. Nkeh-Chungag

**Affiliations:** ^1^Department of Chemistry, Faculty of Science and Agriculture, University of Fort Hare, Private Bag X1314, Alice 5700, South Africa; ^2^Department of Human Biology, Faculty of Health Sciences, Walter Sisulu University, Private Bag X1, Mthatha 5117, South Africa; ^3^Department of Chemical and Physical Sciences, Faculty of Natural Sciences, Walter Sisulu University, Private Bag X1, Mthatha 5117, South Africa; ^4^Department of Biological and Environmental Science, Faculty of Natural Sciences, Walter Sisulu University, Private Bag X1, Mthatha 5117, South Africa

## Abstract

Oleanolic acid is a pentacyclic triterpenoid compound widely found in plants and well known for its medicinal properties. Oleanolic acid (OA) was isolated from the ethyl acetate extract of* Syzygium aromaticum* flower buds. Semisynthesis afforded both acetate and ester derivatives. The derived compounds were monitored with thin layer chromatography and confirmed with nuclear magnetic resonance (NMR) spectroscopy, mass spectrometry (MS), Fourier infrared (FT-IR) spectroscopy, and melting point (Mp). All these compounds were evaluated for their analgesic and anti-inflammatory properties at a dose of 40 mg/kg. Significant analgesic and anti-inflammatory effects were noted for all OA-derived compounds. In the formalin-induced pain test, the derivatives showed better analgesic effects compared to their precursor, whereas, in the tale flick test, oleanolic acid proved to be superior in analgesic effects compared to all its derivatives with the exception of the acetyl derivative. Acute inflammatory tests showed that acetyl derivatives possessed better anti-inflammatory activity compared to the other compounds. In conclusion, semisynthesis of oleanolic acid yielded several derivatives with improved solubility and enhanced analgesic and anti-inflammatory properties.

## 1. Introduction

Plant secondary metabolites have several properties which are useful for the treatment of diseases. Consequently, natural products play an essential role in enhancing modern drug discovery [1–4].

Oleanolic acid (3*β*-hydroxy-olea-12-en-28-oic acid, (OA)) is a well-known pentacyclic triterpenoid isolated from various plants [5, 6]. This compound has shown potent effects against a wide range of pathogens and health conditions [7–9] and has thus held the interest of researchers in the last few decades. OA has shown protection against acute and chronic liver injury [10], breast cancer [11], inflammation [9], and HIV [12].

Despite the many potential uses of OA, it has not been developed into pharmaceuticals due to its instability and low water solubility. Hence, several studies have been designed to modify the structure of OA with the hope of improving its physical properties for better bioavailability to enhance its bioactivity. Moreover, it is clearly demonstrated that the chemical structure of oleanolic acid has three “active” sites, the C_3_ hydroxy, the C_12_ to C_13_ double bond, and the C_28_ carboxylic acid [7, 13, 14], which may be chemically modified and thus change its physical and pharmacological effects.

The work described herein focuses on semisynthesis of oleanane-derived compounds and compares their analgesic and anti-inflammatory effects with those of oleanolic acid, an isolate of* S. aromaticum*.

## 2. Material and Method

### 2.1. Plant Identification

The dried flower buds of* S. aromaticum* were purchased from the spice market in Durban, South Africa, and authenticated in the School of Biological and Conservation Sciences, University of KwaZulu-Natal, Westville Campus. A voucher specimen number 004 was deposited at the University Herbarium.

### 2.2. Plant Preparation and Extraction

Dried flower buds of* S. aromaticum* (L.) were pulverized into fine powder with an electrical food processor. A powdered sample (1.95 kg) was sequentially extracted (twice per solvent) with n-hexane, dichloromethane, ethyl acetate, and methanol, respectively. Each solvent extraction used 1500 mL of the solvent with the clove sample mixture placed on a shaker for five days after which the filtrate was obtained and solvent recovered. Later, 1200 mL of the same solvent was added to the same clove sample as before and extracted on a shaker for two days. A well refined solution was filtered and concentrated with a rotary evaporator and then air-dried at room temperature.

### 2.3. Isolation Method

The ethyl acetate extract (15.535 g) was loaded into a column for column chromatograph using silica gel 60 (0.063–0.200 mm). The column was eluted with a series of solvent mixtures, n-hexane: ethyl acetate (9 : 1, 8 : 2, 7 : 3, 6 : 4, 4 : 6), respectively, and the fractions were visualized on a thin layer chromatography (TLC) plate with anisaldehyde spray reagent. The single spot isolates were characterized for structural elucidation using MS, Mp, FT-IR, and NMR.

The single spot isolate, compound 1 (C_30_H_48_O_3_, OA), was 2.68 g (17.3%) with the following data obtained as a white amorphous powder, Mz+ 456, Mp 294-295°C. FT-IR (ATR, *ν*_max_ cm^−1^): 3404 (-OH), 2941 and 2859 (aliphatic -CH), 1717–1690 (-C=O, COOH), 1465 (-C=C), 1032 (-C-O) respectively. ^1^H-NMR (400 MHz, CDCl_3_): (3H, seven methyl protons, C_23_–C_27_ and C_29_-C_30_) 0.90 s, 0.90 s, 0.89 s, 1.02 s, 1.31 s, 0.91 s, 0.94 s, (1H, five methine proton, C_3_, C_5_, C_9_, C_12_, C_18_) 3.43, 1.35, 1.63, 5.59, 2.47 respectively, (2H, twenty methylene proton, C_1_, C_2_, C_6_, C_7_, C_11_, C_15_, C_16_, C_19_, C_21_, C_22_) 1.38 *α*, 1.53 *β*, 1.89 *α*, 1.64 *β*, 1.58 *α*, 1.59 *β*, 1.38 *α*, 1.79 *β*, 2.19 *α*, 2.18 *β*, 1.84 *α*, 1.74 *β*, 1.95 *α*, 1.92 *β*, 1.45 *α*, 1.55 *β*, 1.44 *α*, 1.55 *β*, 1.78 *α*, 2.08 *β*. ^13^C-NMR (400 MHz, CDCl_3_, C_1_–C_30_): 38.41, 27.66, 78.09, 39.28, 55.29, 18.17, 32.52, 47.55, 37.69, 23.39, 122.65, 143.58, 41.56, 27.66, 22.88, 46.53, 41.63, 45.83, 30.67, 33.79, 32.44, 28.04, 15.38, 16.65, 17.15, 25.90, 182.27, 33.05, 23.57.

## 3. Synthesis of Oleanane-Derived Compounds

### 3.1. 3-Acetyloleanolic Acid (Compound 2) and 3-Triflouroacetyloleanolic Acid (Compound 5)

Compounds 2 and 5 were synthesized using a modified procedure described by Zhu et al. [12]. Oleanolic acid (OA) (0.2 g, 0.4 mmol.) was dissolved in pyridine (5 mL) and acetic anhydride (10 mL) was added in a 150 mL round bottom flask. The mixture was stirred for 12 h at room temperature for compound 2 and 0°C for compound 5. The product was poured into 100 mL of water and stirred for 2 h to hydrolyze excess acetic anhydride. The final product was separated by suction filtration and recrystallized in methanol and, then, purified by column chromatography to give 92% yield of compound 2 (C_32_H_50_O_4_, AOA) and 87% yield of compound 5 (C_32_H_47_O_4_F_3_, TOA).

Compound 2 (C_32_H_50_O_4_, AOA) was a white amorphous powder, Mz+ 498, Mp 265-266°C, FT-IR (ATR, *ν*_max_ cm^−1^): 3205 (-OH, COOH), 2969–2853 (aliphatic -CH), 1723 (-C=O, COOH), 1680 (-C=O, CH_3_COOR), 1457 (-C=C), 1008 (-C-O). ^1^H-NMR (400 MHz, CDCl_3_): (3H, eight methyl singlets) 1.00 s, 1.00 s, 0.91 s, 1.05 s, 1.30 s, 0.92 s, 0.93 s, 2.08 s, (1H, five methine proton, C_3_, C_5_, C_9_, C_12_, C_18_) 4.45, 1.36, 1.60, 5.25, 2.34 respectively, (2H, twenty methylene proton, C_1_, C_2_, C_6_, C_7_, C_11_, C_15_, C_16_, C_19_, C_21_, C_22_) 1.45 *α*, 1.46 *β*, 1.81 *α*, 1.67 *β*, 1.39 *α*, 1.55 *β*, 1.28 *α*, 1.43 *β*, 0.91 *α*, 1.88 *β*, 1.10 *α*, 1.66 *β*, 1.60 *α*, 1.95 *β*, 1.15 *α*, 1.64 *β*, 1.23 *α*, 1.33 *β*, 1.58 *α*, 1.67 *β*. ^13^C-NMR (400 MHz, CDCl_3_, C_1_-C_2′_): 38.07, 27.66, 80.93, 37.69, 55.29, 18.18, 32.44, 39.28, 47.55, 36.98, 23.39, 122.57, 143.59, 41.59, 27.66, 23.57, 46.51, 41.00, 45.84, 30.67, 33.79, 32.43, 28.04, 16.66, 15.38, 17.13, 25.88, 182.76, 33.05, 23.57, 171.05, 21.31.

Compound 5 (C_32_H_47_O_3_F_3_, TOA) was a white crystalline compound, Mz+ 552, Mp 271-272°C, FT-IR (ATR, *ν*_max_ cm^−1^): 3204 (-OH of COOH), 2970 and 2945 (aliphatic -CH), 1771 (-C=O, COOH), 1723 (-C=O, CH_3_COOR), 1455 (-C=C), 1010 (-C-O). ^1^H-NMR (400 MHz, CDCl_3_): (3H, seven methyl protons, C_23_–C_27_ and C_29_-C_30_) 0.95 s, 0.98 s, 0.93 s, 1.15 s, 1.29 s, 1.02 s, 1.32 s, (1H, five methine protons, C_3_, C_5_, C_9_, C_12_, C_18_), 4.73, 1.35, 1.42, 5.30, 2.87 respectively, (2H, twenty methylene proton, C_1_, C_2_, C_6_, C_7_, C_11_, C_15_, C_16_, C_19_, C_21_, C_22_) 1.45 *α*, 1.58 *β*, 1.80 *α*, 1.61 *β*, 1.51 *α*, 1.62 *β*, 1.36 *α*, 1.79 *β*, 2.01 *α*, 2.06 *β*, 1.94 *α*, 1.80 *β*, 1.90 *α*, 1.91 *β*, 1.64 *α*, 1.34 *β*, 1.52 *α*, 1.45 *β*, 1.91 *α*, 175 *β*. ^13^C-NMR (400 MHz, CDCl_3_, C_1_-C_2′_): 39.19, 23.85, 86.89, 37.96, 55.54, 18.11, 33.05, 40.97, 48.05, 36.95, 23.56, 123.69, 143.66, 41.91, 27.87, 23.09, 46.34, 34.76, 45.83, 29.65, 32.48, 32.41, 22.89, 22.38, 15.35, 17.10, 25.90, 187.41, 27.65, 27.26, 157.60, 48.04.

### 3.2. 28-Methyl-3-acetyloleanane (Compound 3) and 28-Methyl-3-trifluoroacetyloleanane (Compound 6)

Compounds 3 and 6 were synthesized using the modified procedure documented by Cheng et al. [15]. Acetyl derivatives of OA (0.2 g, 0.4 mmol) were methylated by iodomethane (2.0 g), then sodium carbonate anhydrous (2.0 g) was added to stabilize the pH, and the whole solution was dissolved in 40 mL dimethylformamide in 150 mL round bottom flask. The solution was stirred overnight at room temperature. The product was poured into 100 mL of water to hydrolyze excess iodomethane and stirred for 2 hrs. The final product was separated by suction filtration and recrystallized in methanol. The comparison with starting material to confirm formation of a new derivative was done with TLC plate, which showed a single spot compound different from the starting material. This method resulted in a 100% yield of compound 3 and 94% yield of compound 6.

Structural elucidation revealed that compound 3 (C_33_H_50_O_4_, AOAm); Mz+ 512, Mp 222-223°C, FT-IR (ATR, *ν*_max_ cm^−1^): 2972–2860 (aliphatic -CH), 1724 (-C=O), 1470 (-C=C), 1020 (-C-O). ^1^H-NMR (400 MHz, CDCl_3_): (3H, nine methyl proton, C_23_–C_27_ and C_29_, C_30_, C_2′_, C_3′_) 1.03 s, 1.02 s, 0.91 s, 1.06 s, 1.11 s, 0.92 s, 0.89 s, 2.03 s, 3.61 s, (1H, five methine protons, C_3_, C_5_, C_9_, C_12_, C_18_) 4.46, 0.81, 1.67, 5.27, 2.82, (2H, twenty methylene proton, C_1_, C_2_, C_6_, C_7_, C_11_, C_15_, C_16_, C_19_, C_21_, C_22_) 1.02 *α*, 1.62 *β*, 1.55 *α*, 1.61 *β*, 1.25 *α*, 1.65 *β*, 1.44 *α*, 1.47 *β*, 1.19 *α*, 2.03 *β*, 1.11 *α*, 1.65 *β*, 1.60 *α*, 1.95 *β*, 1.15 *α*, 154 *β*, 1.49 *α*, 1.57 *β*, 1.58 *α*, 1.67 *β*. ^13^C-NMR: (400 MHz, CDCl_3_, C_1_–C_3′_) 38.11, 27.68, 80.94, 37.69, 55.30, 18.22, 32.38, 39.29, 47.56, 37.69, 23.40, 122.28, 143.81, 41.64, 27.68, 23.52, 46.73, 41.23, 45.85, 30.69, 33.85, 32.60, 28.04, 16.68, 15.36, 18.21, 25.89, 178.32, 33.10, 23.52, 171.03, 21.31, 51.52.

Methylation of compound 5 led to 94% yield of compound 6 (C_33_H_49_O_4_F_3_, TOAm), Mz+ 566, Mp: 283-284°C, FT-IR (ATR, *ν*_max_ cm^−1^): 3437 (-OH), 2940 and 2860 (-CH, aliphatic), 1688 (-C=O), 1461 (-C=C), 1030 (-C-O). ^1^H-NMR (400 MHz, CDCl_3_): (3H, eight methyl protons, C_23_–C_27_ and C_29_, C_30_, C_33_) 1.00 s, 0.94 s, 0.93 s, 1.25 s, 1.27 s, 0.91 s, 0.79 s, 3.25 (1H, five methine protons, C_3_, C_5_, C_9_, C_12_, C_18_), 3.24, 1.37, 1.61, 5.30, 2.82 respectively, (2H, twenty methylene proton, C_1_, C_2_, C_6_, C_7_, C_11_, C_15_, C_16_, C_19_, C_21_, C_22_) 1.54 *α*, 1.46 *β*, 1.92 *α*, 1.86 *β*, 1.57 *α*, 1.67 *β*, 1.42 *α*, 1.78 *β*, 2.03 *α*, 2.04 *β*, 1.85 *α*, 1.72 *β*, 1.79 *α*, 1.83 *β*, 1.58 *α*, 1.57 *β*, 1.43 *α*, 1.49 *β*, 1.39 *α*, 144 *β*. ^13^C-NMR (400 MHz, CDCl_3_, C_1_–C_3′_): 38.67, 23.57, 79.03, 38.75, 55.21, 17.13, 33.06, 39.27, 47.63, 37.08, 23.40, 122.64, 143.59, 41.61, 28.10, 27.68, 45.87, 41.00, 45.88, 32.43, 33.80, 33.06, 22.93, 23.57, 15.31, 15.53, 25.93, 182.87, 28.10, 27.18.

### 3.3. 28-Methyloxyoleanolic Acid (Compound 4)

The modified method of Mallavadhani et al. [11] was employed during the experiment. OA (0.2 g, 0.4 mmol.) was dissolved in acetone (2 mL) and then anhydrous K_2_CO_3_ (0.1 g) and CH_3_I was added dropwise with constant stirring at room temperature. The mixture was constantly stirred for 12 h at room temperature; then, the whole solution was diluted with 100 mL of water and stirred for 2 hrs. The whole solution was further extracted with chloroform and the organic layer dried in anhydrous sodium sulphate at room temperature. A single spot compound was obtained and structurally elucidated with FT-IR, MS, Mp, and NMR.

White crystalline compound 4 (OAm, C_31_H_50_O_3_, OA) was obtained at 98% yield, Mz+ 470, Mp: 139-140°C, FT-IR (ATR, *ν*_max_ cm^−1^): 3346 (-OH), 2940 (-CH), 1728–1710 (-C=O), 1462 (-C=C), 1032 (-C-O). ^1^H-NMR (400 MHz, CDCl_3_): (3H, eight methyl protons, C_23_–C_27_ and C_29_, C_30_, C_33_) 0.95 s, 0.90 s, 0.88 s, 1.02 s, 1.31 s, 0.92 s, 0.84 s, 3.60 (1H, five methine protons, C_3_, C_5_, C_9_, C_12_, C_18_), 3.20, 1.35, 1.58, 5.26, 2.82 respectively, (2H, twenty methylene proton, C_1_, C_2_, C_6_, C_7_, C_11_, C_15_, C_16_, C_19_, C_21_, C_22_) 1.42 *α*, 1.45 *β*, 1.67 *α*, 1.62 *β*, 1.55 *α*, 1.61 *β*, 1.38 *α*, 1.64 *β*, 2.02 *α*, 1.98 *β*, 1.90 *α*, 1.66 *β*, 1.91 *α*, 1.94 *β*, 1.49 *α*, 1.53 *β*, 1.47 *α*, 1.51 *β*, 1.84 *α*, 1.98 *β*. ^13^C-NMR (400 MHz, CDCl_3_, C_1_–C_3′_): 38.44, 27.19, 79.03, 38.76, 55.22, 18.33, 32.66, 39.27, 47.64, 37.04, 23.41, 122.36, 143.79, 41.65, 27.71, 23.64, 46.73, 41.30, 45.89, 30.70, 33.86, 32.39, 28.10, 15.57, 15.30, 16.84, 25.94, 178.32, 33.11, 23.08, 51.53.

## 4. Bioassays

### 4.1. Experimental Animals

Wistar rats (180–250 g) and Swiss mice (20–35 g) of either sex were used for bioassays. These animals were housed with day light being the only source of lighting and had free access to food and water. Animals were fasted overnight before experimentation. Ethical clearance for this study was obtained from the Walter Sisulu University Ethics Committee (DVC (AA&R) DRD/SREC: reference number 31) in accordance with the ethical standards laid down by the Declaration of Helsinki (2000). All drugs and compounds were prepared in normal saline and administered orally.

### 4.2. Formalin-Induced Pain Test

The formalin test was carried out as described by Jang et al. [16] with some modifications. Eight groups of mice (six mice per group) were treated with ibuprofen (100 mg/kg), OA, and its derived compounds (40 mg/kg) while control animals were treated with normal saline. One hour later, 50 *μ*L of 1% v/v formalin was injected in the right hind paw of animals. Animals responded by licking/biting the injected paws. The number of paw licking/bites were counted during the first 5 minutes (neurogenic phase) and then during the 10–30 minutes (inflammatory phase) after formalin injection.

### 4.3. Tail Flick Test

The method used by Nkomo et al., [17] was used for the tail flick test. Each treatment group constituted 6 animals. Baseline tail flick latencies were obtained for each animal using the Ugo Basile Tail Flick Machine (model 37360), after which drugs were administered orally at a dose of 40 mg/kg for OA and its derivatives and 100 mg/kg for ibuprofen and control was treated with normal saline. Tail flick latencies were again assessed hourly for 5 h after drug administration.

### 4.4. Albumin-Induced Inflammation

Baseline right hind paw volume was measured using the Ugo Basile plethysmometer as described by Iannitti et al. [18]. This was followed by oral treatment of animals as per assigned group with 40 mg/kg of all compounds or ibuprofen (100 mg/kg) while control animals were treated with 0.09% of NaCl. Thirty minutes after treatment, inflammation was induced by injection of fresh egg-albumin (0.1 mL, 50% v/v in saline) into the right hind paw. Paw volumes were determined plethysmographically at 30 min and 1, 2, 3, and 4 hours after injection of the phlogistic agent.

### 4.5. Statistical Analysis

GraphPad Instat® was used to analyze all results. ANOVA followed by Dunnets test was used to determine differences in treatment groups. Unpaired *t*-test was used to compare means of treated groups with the mean of the control group. A second analysis comparing mean of results obtained with OA treatment with results obtained with OA derivatives was performed. Results were expressed as mean ± SE where *n* = 6; *p* < 0.05 was considered significant.

## 5. Results

### 5.1. Oleanane-Derived Compounds

**Figure 1:**
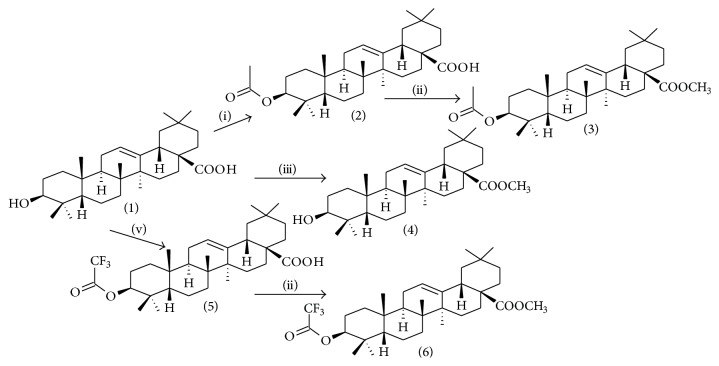
Semisynthetic pathways: (i) (CH_3_CO_2_)_2_O, pyridine, 12 hrs, 25°C, (ii) CH_3_I, DMF, 12 hrs, 25°C, (iii) K_2_CO_3_, CH_3_I, 12 hrs, 25°C, and (v) (CF_3_CO_2_C)_2_O, pyridine, 12 hrs, 0°C.

Modification of oleanolic acid (OA) led to acetate and ester derivatives. OA-derived compounds were synthesized as illustrated in Figure 1. Acetyl-OA derivatives, compounds 2 and 5, were obtained with 87% and 92% yields, respectively. These compounds resulted from the decoration of the C_3_ atom of OA in dry pyridine with acetic anhydride at 25°C for compound 2 and trifluoroacetic anhydride at 0°C for compound 5 [12]. For ester derivatives, compounds 3, 4, and 6, were obtained with yields of 100%, 93%, and 94%, respectively. These were formulated by introducing a methyl group as illustrated in Figure 1 [11, 15].

### 5.2. Functional Group Comparison of Oleanane-Derived Compounds with Oleanolic Acid

FT-IR of oleanolic acid (compound 1, OA) and its derivatives is illustrated in Table 1. The formation of new functional groups on targeted position of the chemical structure of compound 1 was successfully elucidated. In Table 1, OA discloses the following major functional groups at 3406 cm^−1^ (-OH) stretching of a free alcohol at C_3_, 2835, 2864 cm^−1^ aliphatic (-CH) stretching of an alkane, 1688 cm^−1^ (-C=O) bending of a carboxylic acid at C_28_, and 1460 (C=C) stretching of alkene group at C_12_ of the chemical structure. However, acetylation of OA at position C_3_ resulted in the formation of an acetyl group which was confirmed by FT-IR spectroscopy. Acetyl derivatives of OA (compounds 2 and 5) are illustrated in Table 1; compound 2 shows an absorption band at 3205 cm^−1^ (-OH, carboxylic acid), 2969–2853 cm^−1^ (stretch of aliphatic -CH), 1723 (-C=O, carboxylic acid), 1680 (-C=O, acetate), and 1457 (-C=C, alkene). Compound 5 shows almost similar peaks to those of compound 2 at 3204 cm^−1^ (-OH of carboxylic acid), 2970, and 2945 cm^−1^ (aliphatic -CH stretch) and two absorption bands at 1771 cm^−1^ (-C=O, carboxylic acid), 1723 cm^−1^ (-C=O, acetate), and 1455 (-C=C, alkene). In both compounds, the free -OH of an alcohol at C_3_, ring A of the chemical structure of OA, was successfully acetylated. In addition, the methylation of C_28_, ring E of an acetyl derivative of OA compound, led to the formation of two ester derivatives, that is, compounds 3 and 6.

Compound 4 28-methyloleanane was derived directly from OA. Compound 4 was obtained after treating OA with iodomethane and then K_2_CO_3_ was added to stabilize the pH. FT-IR shows carbonyl shift compared to that of OA; this confirms the formation of a methyl ester in the place of carboxylic acid.

### 5.3. NMR Interpretation

Full house NMR (^1^H, ^13^C, DEPT, COSY, NOESY, HSQC, and HMBC) analysis was employed to successfully elucidate the chemical structure of oleanane-derived compounds. The significant difference between compound 1 (oleanolic acid, OA) and its derivatives was obtained by comparison of the spectra. Compound 2 (3-oleanolic acid, AOA) spectrum differs from that of OA with a prominent singlet at 2.04 ppm (**CH**_**3**_COO) attached at C_3_ position of the chemical structure. The effect of acetyl group causes a deshielding of a doublet at C_3_ from 3.43 to 4.45 ppm.

Moreover, methylation of AOA at C_28_ results in compound 3 (28-methyl, 3-acetyloleanane, AOAm) and its ^1^H-NMR spectrum discloses a noticeable singlet at 3.61 ppm (**CH**_**3**_ **O**OC-). The ^1^H-NMR spectrum of AOAm differs with two singlets (2.03 ppm for acetate and 3.61 ppm for an ester) from OA spectrum. A direct methylation of OA results in compound 4 (28-methyloleanane, OAm) and the ^1^H-NMR displayed a prominent singlet at 3.47 ppm due to the formation of an ester (**CH**_**3**_ **O**OC-) from carboxylic acid group.

Compound 5 (3-trifluoroacetyloleanolic acid, TOA) was obtained by acetylation of OA with trifluoroacetic anhydride. A singlet of singlet was obtained from ^1^H-NMR spectrum at 2.00 and 2.01 ppm (**CF**_**3**_ **C**OO-). The TOA differs only with that signal at C_3_ position compared with OA. However, methylation of TOA has led to 28-methyl, 3-trifluoroacetyloleanane (TOAm). The ^1^H-NMR of TOAm discloses a methyl proton at 1.79 ppm (**CH**_**3**_ **O**OC-). The formation of methyl- group at position 28 has led to a shift (shielding) of a proton on position 3 of chemical structure to the upfield 3.24 ppm. The methyl group signal was expected between 2.5 and 3.5 ppm; however, the synthetic route used in this study influences the signal to shield to upfield 1.79 ppm. Secondly, trifluoroacetyl functional group which is more acidic affects the nucleus of methyl group at position 28 to “feel” weak magnetic field. However, methylation of TOA led to compound 6 (28-methyl, 3-trifluoroacetyloleanane, TOAm). The ^1^H-NMR spectrum of TOAm discloses a methyl proton at 1.76 ppm (**CH**_**3**_ **O**OC-) and all this information was supported by DEPT, COSY, NOESY, HSQC, and HMQC-NMR spectra.

Therefore, the following chemical formulas are confirmed: C_32_H_50_O_4_ for compound 2 (AOA), C_33_H_52_O_4_ for compound 3 (AOAm), C_31_H_50_O_4_ for compound 4 (OAm), C_32_H_47_F_3_O_4_ for compound 5 (TOA), and C_33_H_50_F_3_O_4_ for compound 6 (TOAm) with support of MS.

## 6. Biological Test

For ease of report, the biological effects of acetyl and trifluoroacetyl compounds were presented separately.

### 6.1. Analgesic Effects of OA and Derivatives: Tail Flick Test

**Figure 2:**
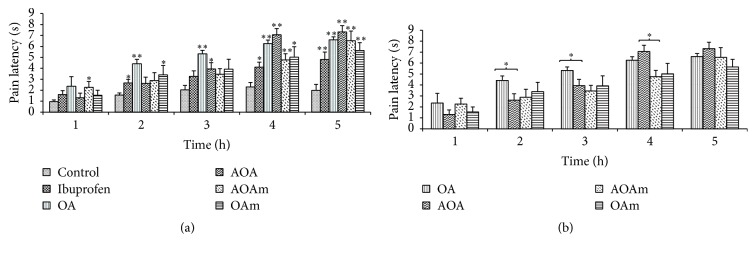
Analgesic effect of OA and its acetyl derivatives on tail flick response induced by radiant heat. OA (oleanolic acid), AOA (3-acetyloleanolic acid), AOAm (28-methylester, 3-acetyloleanane), and OAm (28-methyloleanane). Values are presented as mean reaction time in seconds ± SEM for 6 animals; ^*∗*^ *p* < 0.05 and ^*∗∗*^ *p* < 0.01. (a) shows a comparison of the tested compounds with control group, whereas (b) shows comparison of the OA with acetyl derivatives of OA.

Figure 2(a) depicts time dependent analgesic effects of treatments in response to radiant pain. The effect of all compounds used was time dependent though AOAm had an early onset of analgesic effect (1 h) compared to the other compounds. Pain latency to radiant heat was significantly (*p* < 0.01) increased in all drug treated animals at 4 and 5 h after treatment.  Figure 2(b) compared OA derivative treatment groups with the OA treated group. Unpaired *t*-test with two-tailed *p* values showed that OA increased pain latency significantly (*p* < 0.05) during the 2 and 4 h test periods compared to AOA. 3-Acetyloleanolic acid (AOA) inhibited radiant heat-induced pain significantly and showed better analgesic activity compared to OA between 4 and 5 h after treatment. The analgesic effects of OA increased rapidly from 1 to 3 h showing significantly higher pain latency at 2 h compared to AOA though the effects of the latter became significantly greater than those of OA during the 4 h.

**Figure 3:**
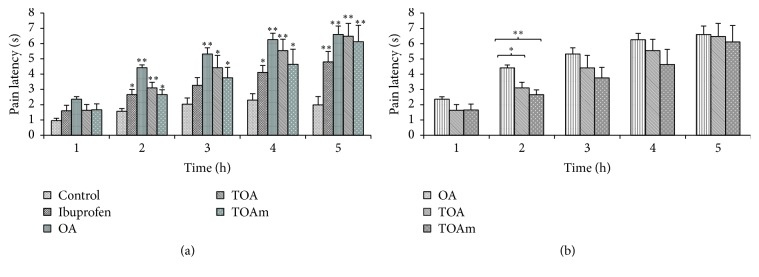
Effect of trifluoroacetyl derivatives of OA on pain latency to radiant heat and of OA (oleanolic acid), TOA (3-trifluoroacetyloleanolic acid), TOAm (28-methylester, 3-trifluoroacetyloleanane), and ibuprofen (standard drug) on latency to radiant heat. Values are presented as mean reaction time in seconds ± SEM for 6 animals; ^*∗*^ *p* < 0.05 and ^*∗∗*^ *p* < 0.01. (a) shows a comparison of the tested compounds against control group, whereas (b) shows the comparison of the semisynthesized trifluoroacetyl compounds with OA.

The analgesic effects of OA and its trifluoroacetyl derivatives increased with time with best effects noted during the 5 h after treatment. All treatment groups had significantly (*p* < 0.05 or *p* < 0.01) increased reaction times to radiant heat (Figure 3(a)). OA had a noticeable earlier onset of pain inhibition (1 h); its effects however were significantly better than those of the trifluoroacetyl derivatives only during 2 h after treatment. Beyond this time, the analgesic effects of OA were very similar to those of its derivatives (Figure 3(b)).

### 6.2. Analgesic Effects of OA and Derivatives: Formalin Test

**Figure 4:**
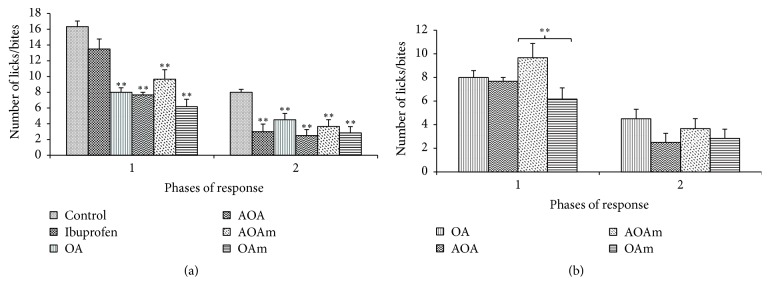
Formalin-induced pain behavior test. OA (oleanolic acid), AOA (3-acetyloleanolic acid), AOAm (28-methylester, 3-acetyloleanane), and OAm (28-methyloleanane). Results are presented as mean number of licks/bites ± SEM for 6 animals; ^*∗*^ *p* < 0.05 and ^*∗∗*^ *p* < 0.01. (a) shows a comparison of the tested compounds with control group, whereas (b) shows the comparison of the oleanane derivatives with OA.

The formalin-induced pain test generated the classical biphasic response which is characterized by flinching or licking/biting of injected paw. OA and all its acetyl derivatives significantly (*p* < 0.01) reduced the number of paw licks/bites in both the first and second phases of the experiments (Figure 4(a)). When results were individually compared with results from the OA treated group, the analgesic effects of OA were not significantly different from those obtained with OA derivatives. However, AOAm treatment seemed to significantly increase the pain threshold in the first phase of the tests (Figure 4(b)).

**Figure 5:**
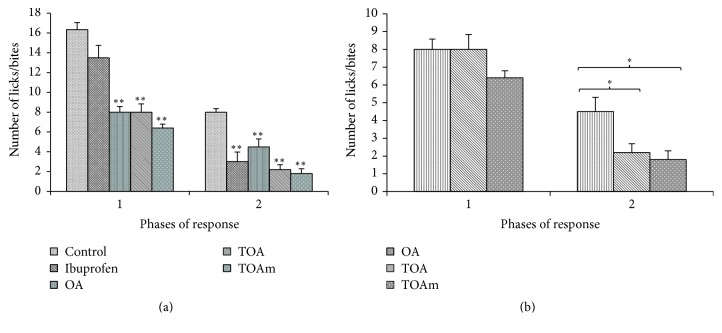
Formalin-induced pain behavior. OA (oleanolic acid), TOA (3-trifluoroacetyloleanolic acid), and TOAm (28-methylester, 3-trifluoroacetyloleanane). Results are presented as mean number of licks/bites ± SEM for 6 animals; ^*∗*^ *p* < 0.05 and ^*∗∗*^ *p* < 0.01. (a) shows a comparison of the tested compounds with control group, whereas (b) displays a comparison of the trifluoroacetyl derivatives with OA.

Figure 5 shows that OA and its trifluoroacetyl derivatives reduced the number of paw licks/bites in the neurogenic and inflammatory phases of the formalin test. Ibuprofen had a very weak pain inhibitory effect during the first phase though its effects became significantly greater during the second phase of the formalin test (Figure 5(a)). A comparison of response to formalin-induced pain behavior in trifluoroacetyl derivatives of OA with the parent molecule, OA, showed that the latter had significantly weaker analgesic effects compared with TOA and TOAm, respectively, during the second phase of the test (Figure 5(b)).

### 6.3. Anti-Inflammatory Effects of OA and Derivatives

**Figure 6:**
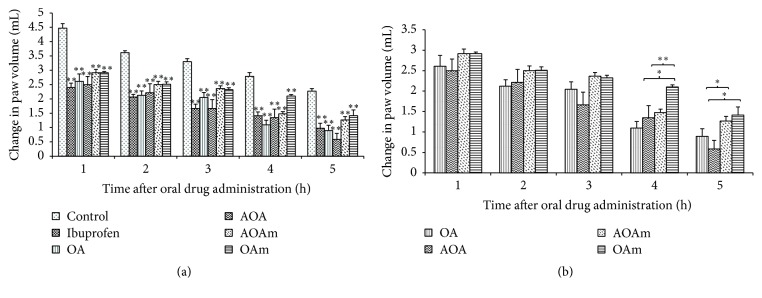
Anti-inflammatory effect of OA (oleanolic acid), AOA (3-acetyloleanolic acid), AOAm (28-methyl, 3-acetyloleanane), and OAm (28-methyloleanane). Results are presented as mean change in paw volume ± SEM for 6 animals; ^*∗*^ *p* < 0.05 and ^*∗∗*^ *p* < 0.01. (a) shows a comparison of the tested compounds with control group, whereas (b) shows the comparison of anti-inflammatory effects of the oleanane compounds with OA.

The anti-inflammatory effects of all test compounds increased with time as depicted by the changes in paw volume compared to baseline volumes. All tested compounds significantly (*p* < 0.01) inhibited the inflammatory response to injected albumin (Figure 6(a)). However, a comparison of the anti-inflammatory effects of OA with its acetyl derivatives showed that its effects were significantly (*p* < 0.01) better than those of OAm during the 4 and 5 h after treatment.

**Figure 7:**
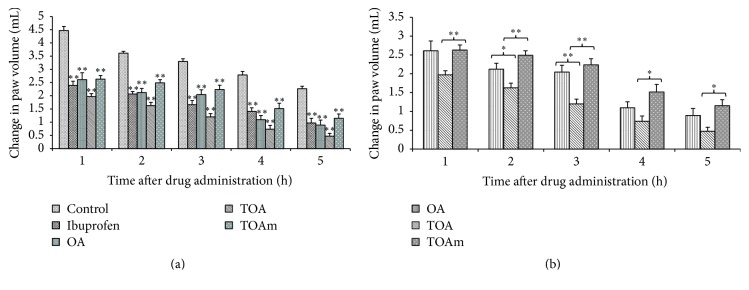
Anti-inflammatory effect of the trifluoroacetyl derivatives of OA. Results are expressed as mean change in paw volume ± SEM for 6 animals; ^*∗*^ *p* < 0.05 and ^*∗∗*^ *p* < 0.01. (a) shows a comparison of the tested compounds with control group, whereas (b) shows the comparison of anti-inflammatory effects of the trifluoroacetyl derivatives of OA with OA.

Trifluoroacetyl derivatives of OA also showed significant (*p* < 0.01) anti-inflammatory effects (Figure 7(a)). On the other hand, the anti-inflammatory responses of TOA treated animals were significantly (*p* < 0.05 and 0.01) better than the effects of both OA and TOAm treatments, respectively (Figure 7(b)).

## 7. Discussion

Oleanolic acid possesses multiply pharmacological properties which include anti-inflammatory, antitumor, and analgesic properties [19, 20]. Recently, OA was documented as a promising lead compound for new drug formulation [21]. Indeed, Habila et al. [22] demonstrate the improvement of antibacterial effect of OA by decorating C_3_ position hydroxy group with an acetyl group while Nkeh-Chungag et al. [14] demonstrated improved analgesic and anti-inflammatory properties by semisynthesis of OA. The present study corroborates the fact that modification of OA in C_3_ and C_28_ positions results in enhancement of biological properties. Furthermore, all oleanane-derived compounds also display superior analgesic activity in formalin-induced pain [23]. The formalin test is a useful nociceptive model in that pain is spontaneous and responses are observed in freely moving animals. This pain closely resembles pain response to injury in humans. The first phase generally referred to as the neurogenic phase occurs during the first 5 minutes after formalin injection while the second phase or inflammatory phase occurs during the 10–30 minutes after formalin injection [21].

The neurogenic phase of the formalin test involves the direct chemical stimulation of nociceptors while the inflammatory phase involves the release of mediators such as prostaglandins, serotonin, histamine, and bradykinin [24]. Tsai et al. [25] and Checker et al. [26] demonstrated that OA is less active in the neurogenic phase whereas it shows exceptional inhibition in the inflammatory phase of the formalin-induced pain. Indeed, our results showed strong pain inhibitory effects of OA during the first and second phases of the formalin-induced pain test. Both the acetyl and trifluoroacetyl derivatives of OA showed a tendency for better analgesic effects. However, only TOA and TOAm were significantly better analgesic agents than OA during the second phase indicating the influence of trifluoroacetyl group on the properties of OA. These results indicated that trifluoroacetyl derivatives may have a better anti-inflammatory effect than OA. Indeed, trifluoroacetyl decorated derivatives of OA tended to inhibit inflammation better than OA.

## 8. Conclusion

Decorated derivative of OA (28-methyloleanane, 28-methyl-3-acetyloleanane (3), and 28-methyl-3-trifluoroacetyloleanane) showed enhanced analgesic and anti-inflammatory properties compared to the parent molecule OA.

## Figures and Tables

**Table 1:** Interpretation of FT-IR spectrum of oleanane-derived compounds.

Compounds	Absorption bands				
	*ν*_C-OH_	*ν*_C-H_	*ν*_C=O_	*ν*_C=C_	*ν*_C-O_
1	3406	2935, 2864	1688	1460	1034
2	3205	2969–2853	1723, 1680	1457	1008
5	3204	2970, 2945	1771, 1723	1455	1010
3	—	2972, 2966	1724	1470	1020
6	—	2940, 2860	1688	1461	1030
4	3346	2940	1726–1710	1462	1031

C_3_, in ring A of the chemical structure of OA, was successfully acetylated. In addition, the methylation of C_28_, ring E of an acetyl-derivative of OA compound, led to formation of ester derivatives: compounds 3 and 6. Compound 4 is 28-methyloleanane derived directly from OA by treating OA with iodomethane after which K_2_CO_3_ was added to stabilize the pH. FT-IR shows carbonyl shift compared to that of OA; this confirms the formation of a methyl ester in the place of carboxylic acid.
